# The determinants of a resilient food system for Finland in the 2020s—three opinion polls for improvements based on a Delphi study among food system experts

**DOI:** 10.1186/s40309-023-00215-z

**Published:** 2023-03-13

**Authors:** Pasi Rikkonen, Karoliina Rimhanen, Kalle Aro, Jyrki Aakkula

**Affiliations:** Natural Resources Institute Finland (Luke), Bioeconomy and Environment, Latokartanonkaari 9, 00790 Helsinki, Finland

**Keywords:** Cluster analysis, Delphi method, Food system, Resilience

## Abstract

Sudden shocks, disturbances, and changes in today’s and the future’s operating environment call for a more resilient food system. COVID-19 and Russia’s latest war in Ukraine have revealed that rapid shocks and disturbances in global social-ecological systems can affect societies and pose various risks to food security. The discussion of food security and especially food supply security under uncertain changes has therefore been highlighted. In this study, food system resilience and the driving forces and current trends affecting it were evaluated in an expert panel study. We used the Delphi technique for the data collection, which included several rounds of iterative evaluation of future food system development up to 2030. We analysed the results using cluster analysis. Based on the results, three clusters were identified which weigh different points within the development foci of food system resilience, namely: (1) the lack of efficient and consensual global crisis preparedness, (2) looking after domestic food production, and (3) trusting the current good efforts in resilience building. The key resilience determinants in each cluster and their importance analysis are presented. We also present enablers and barriers from techno-economic, politico-institutional, and socio-cognitive perspectives that hinder or support resilience building.

**Supplementary Information:**

The online version contains supplementary material available at 10.1186/s40309-023-00215-z.

## Highlights

• The weakest point in terms of food system resilience is primary production and its poor profitability.

• Consensus topics increased protein crop and domestic RE production and the backup power systems.

• Collaboration between actors increases trust and systemic understanding of food system development.

• Clear targets and criteria for food system resilience should be created.

• Resilience should be included in the strategies that guide food system development.

**Supplementary Information:**

The online version contains supplementary material available at 10.1186/s40309-023-00215-z.

## Introduction

The food system faces various social, economic, and ecological driving forces, changes, trends, and disruptions. The need for holistic management has been acknowledged among actors and society. COVID-19 has revealed how vulnerable the global food systems are to disruptions, highlighting the importance of resilience for food security [1, 2]. In Finland, as well as elsewhere in Europe, the major impacts of the COVID-19 pandemic on agriculture and food markets were faced through disruptions to the free movement of employees, causing labour shortages. Export restrictions imposed by some countries have also disrupted trade flows for staple foods [3]. Increasing the understanding of critical factors and means to enhance food system resilience is vital for creating tools for stakeholders to manage resilience and support policymaking in advancing food system resilience.

Food systems are under pressure to transform into operating within planetary boundaries, adapting to climate change and dietary changes, and cutting emissions. The projected increase in food and nutrition insecurity on a global scale has been driven by different shocks and stressors that often overlap or interact. They can be categorised into the following four clusters [4] with some illustrative examples: (1) climate change, variability, and extremes (e.g. erratic rainfall, droughts), (2) conflict and insecurity (e.g. displacement, civil unrest, terrorism), (3) economic downturns and market disruptions (e.g. the food price spikes of 2008); and (4) other unexpected shocks (e.g. the sudden outbreak of desert locusts or a pandemic).

The coronavirus pandemic has increased uncertainty, disrupted production chains, caused a labour shortage threat, and led to drastic measures in several countries to limit the spread of the virus while securing economic recovery after the crisis [3]. The coronavirus triggered a demand shock in the food market, which occurred as a shift in demand to grocery stores and long-life basic foodstuffs in the spring of 2020 [5]. Nevertheless, food prices have remained relatively stable despite the pandemic and have been steadily increasing since January 2018. The main concern in primary production has been the lack of profitability. The trend in profitability has been declining in both agriculture and horticulture throughout the 2000s. This development is due to poor producer price development, price fluctuations, and increasing input prices [6].

**Fig. 1:**
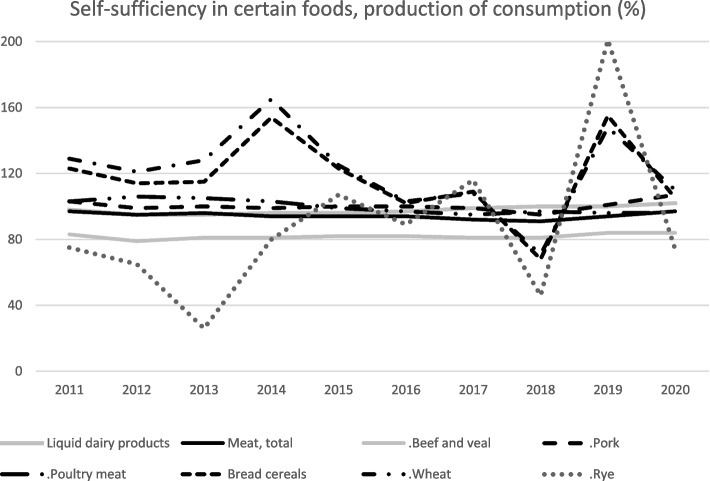
Food self-sufficiency in Finland [7]. Self-sufficiency in certain foods, production of consumption (%)

During the 2 years of the pandemic, security of supply and self-sufficiency have been much discussed in Finland. Finland is almost self-sufficient in basic foodstuffs. The production volume of pork and poultry meat has covered domestic demand in recent years, while beef production accounts for around 80% of its consumption. Moreover, the self-sufficiency rate of Finnish milk production, calculated according to milk protein, still significantly exceeds 100% [5]. Yields produced in the Finnish total field area enable a high self-sufficiency in basic foodstuffs in normal conditions (see Fig. 1).

Changes in food consumption patterns change slowly. However, the discussion of change in diets of a more plant-based and fish-based diet has received much attention in Finland. The dietary change will probably revolutionise the agricultural and food sector in the long run. That said, the total output of agriculture is anticipated to be the same as today only if production still contains at least some meat production [8]. Between 2011 and 2020, there have not been drastic changes in the food sector’s total output [9]. However, growth is expected in fishing and aquaculture and in nature tourism and recreation, which is tightly connected to food services (not included in the food sector’s total output). The indications remain. For the first time ever, the consumption of domestic meat showed signs of a decrease in 2020 [5]. This almost exclusively concerns pork. The domestic consumption of beef remains relatively stable, and poultry meat consumption is growing strongly. A decrease in the consumption of liquid milk has also occurred [5]. Uncertainty may also arise in the availability of imported products and inputs that primary production needs.

It is argued that a controlled change to a sustainable food system requires new value chains and investments to produce plant-based products [8]. This calls for a future-oriented approach to show the possible, feasible, and desirable transition paths to the future. In addition to the sustainability transition, ensuring the food supply is the most important task of the food systems, including during exceptional circumstances like a pandemic or geopolitical conflict. This calls for resilience thinking in food system development among the other objectives. It is therefore important to identify which factors and issues affect the availability of food, thereby strengthening resilience, and anticipate how the food system is changing from a long-term perspective. Tendall et al. [10] define food system resilience as the ‘capacity over time of a food system and its units at multiple levels, to provide sufficient, appropriate and accessible food to all, in the face of various and even unforeseen disturbances’. In this study, we follow the holistic definition by Tendall et al. [10] according to which a resilient food system is able to buffer, persist, adapt, and transform if required under uncertainty to maintain food security [10]. Meuwissen et al. [11] identified three capacities of resilience: robustness, which is related to a system’s capacity to resist and withstand change, adaptability, which refers to a system’s ability to adjust its operations in response to change,and transformability, which is the capacity to change internal structures and operations in response to change. In recent years, resilience studies have been published extensively [10, 12, 13, 14]. Especially, COVID-19 and other recent shocks have revealed vulnerabilities in the food system (e.g. [1, 2, 15, 16, 17]).

In this paper, based on food system expert views, we analyse the key determinants of a resilient food system. Identifying and developing the key determinants can prevent situations of sudden food shortages caused by different shocks like COVID-19. The study forms a practice-oriented view of the food system actors’ roles in making food systems’ foresight more efficient when confronting changes. The paper has three research questions:

1. What are the key priorities for the development of a resilient food system for the Finnish food system expert community?
2. How do key priorities and weights vary among food system expert community?
3. What are the enablers promoting and barriers hampering the resilience of the Finnish food system?

The paper is constructed as follows: first, the background of the study is presented from the perspective of resilience and the future scenarios of food systems; second, the method and data, namely the Delphi method, are presented; third, the results from the Delphi study are analysed, and special attention is paid to the differences and emerging topics within the questions and future views; and finally, the results are discussed, and conclusions are drawn, in the light of the literature from food systems’ resilience and future scenarios.

## Material and methods

We use an expert-based Delphi method in this study. The Delphi method consists of the judgement of experts by means of successive iterations of given topics to show a possible convergence of opinions and identify dissent or non-convergence [18]. The Delphi method is considered as one of the most used methods in the field of foresight, especially for long-range studies (20 to 30 years) (see [18, 19]). Keeney et al. [20] state that the Delphi technique has much to offer in terms of gaining consensus from a wide range of individuals on specific topics. However, they call for a critical review of the Delphi as a robust and systematic approach to data collection. Keeney et al. [20] are especially concerned about how Delphi applications use sampling, anonymity, use of experts, rounds, and application.

**Fig. 2:**
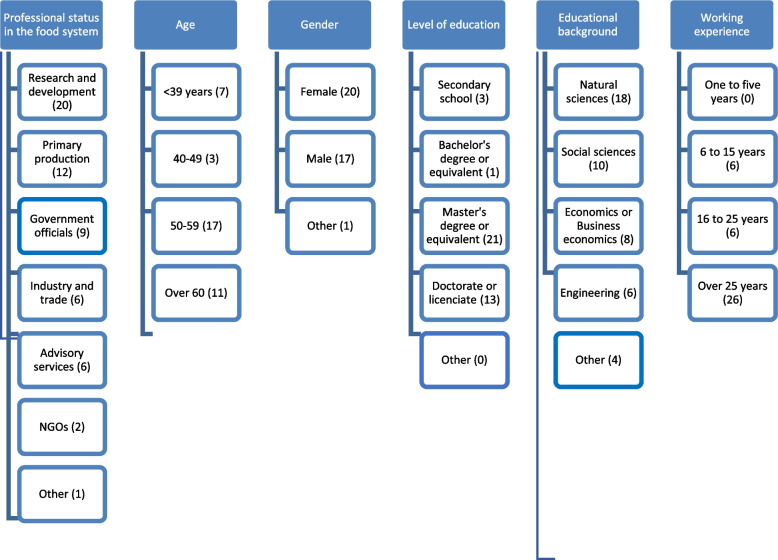
The expertise of the Delphi panel

A random sample of respondents was not used in this study. Instead, the Delphi expert panel included experts from all major food system actors, from primary production to the input and processing industry, retail, and support systems such as research, governance, policymaking, and advisory as the target group of this work. The participants and their background for the future evaluation of the determinants of a resilient food system can be seen in Fig. 2 (note that an expert can have several professional and educational backgrounds).

According to Kuusi [21], the method for selecting the Delphi expert panel is one of the most critical phases of a Delphi study. In their actor analysis, the Delphi facilitator should consider the most important stakeholders and interest groups, the most important substance (the competence of experts), and the terms for delivering information in a Delphi process. The selection process of an expert panel should be done as explicitly as possible. According to Kuusi [21], the information policies depend on three kinds of interacting factors: the personal competencies of the expert, the norms of the respondent’s organisation, and the organisers of foresight studies. The reason for establishing an expert panel is to get the best possible information as bases for preparing strategies and subsequently decisions, in this case meaning defining determinants, analysing interdependences, and improving the base of resilience. Generally, the Delphi process can involve from 10 to several hundreds or even thousands of respondents in the panel [19, 21, 22].

The anonymity principle was followed during the Delphi round questionnaires. Anonymity provides an equal chance for each panel member to present and react to ideas unbiased by the identities of other participants [23]. However, complete anonymity cannot be guaranteed when using this method. As Keeney [20] explains, researchers involved know the panel members and their responses, and it is often the case that panel members know each other. In this study, a Webropol online survey tool was used, and an individual panel member cannot attribute responses to any other member.

Keeney et al. [20] states that number of rounds depends upon the time available and whether the experimenter commenced the Delphi sequence with one broad question or with a list of questions or events. These are usually from two to four rounds in a Delphi process (see [24, 25]). To define the determinants of resilience and therefore the key foci of food system foresight, we used a three-step expert evaluation process. The first step conducted by interviews defined determinants of resilience and food system foresight with food system actors close to the national security of supply [26]. A total of nine experts were interviewed. This step sets the scene and relevant questions for the following two Delphi rounds. McKenna [27] states that using face-to-face interviews in the first-round increases the involvement and motivation of panel members, and therefore, the return rates of questionnaires also increase. Based on step 1 interview results, step 2 proposed statements about the current food system’s resilience and therefore tested the current determinants in an online survey. A total of 38 food system experts responded (see Fig. 2). Step 2 also constructed probable and preferred future views of the Finnish food system and identified the needs for improvement, but this is analysed in another paper [28]. The step 3 data, in which the prioritisation of the key measures for enhancing food system resilience [29], are not utilised here.

### Principles of cluster analysis

The Delphi survey data were analysed using the IBM SPSS statistical program. The cluster analysis was used to categorise the varying expert views on asked resilience statements. Initially, the run of descriptive statistics was conducted to obtain an overview of the valid and missing values. The missing values were then recoded in the same variables, and these values were then replaced with the series mean. Cluster analysis is an exploratory method [30]. It has been used in foresight, especially in the construction of scenarios using the Delphi technique in the agricultural and forestry sectors, the energy sector, climate and energy policy, and the traffic sector [22, 31, 32, 33]. Hierarchical cluster analysis is an agglomerative method, and it starts with individual samples and forms a cluster of the most similar samples, progressively joining observations and clusters until all observations have been joined into a single large cluster in the final stage. In this study, the methodological process of Varho and Tapio [34] in cluster analysis was followed, i.e. used data analysis consists of Delphi process, cluster analysis of numerical material, and qualitative content analysis of open-phrased questions, and as a summary, a futures table is presented (see also disaggregative policy Delphi application in [31]. Cluster analysis simplifies the variance within the data. Some three to seven alternative future states have often been considered reasonable (e.g. [32]).

The cluster validation was done by (1) comparing the results of two different sets of cluster solutions (three- and four-cluster solutions) to determine which is better and (2) proximity matrix that can evaluate the ‘goodness’ of the clustering by looking at the correlations between observation pairs. This also helped in determining the interpreted ‘correct’ number of clusters. The proximity matrix is presented in Additional file 1: Appendix 1.

Each of the sections in the survey also included open-ended questions. The responses were used to deepen the content of the cluster analysis. The categorisation of the open-ended responses did not follow the clusters. Instead, we sought the most illustrative arguments to enrich each cluster-based narrative.

## Results

The results consist of three sections from the Delphi survey process: (1) the statements of the determinants, drivers, and changes of a resilient food system; (2) the stated importance of the disruptions, drivers, and changes the food system faces; and (3) open-ended answers that were asked after “Introduction” and “Material and methods” sections (the respondent could make further arguments based on his/her view of the questions).

Several runs with the dataset were conducted to test how the data reacted to *K*-means and hierarchical clustering algorithms and which number of chosen clusters provided a relevant basis for interpretations of the Delphi data. *K*-means clustering was abandoned because it does not allow clusters to form freely. Instead, you must select how many clusters there are. In hierarchical cluster analysis, you can either decide beforehand the number of clusters or let them form freely. As such, cluster analysis is an iterative process where researcher’s subjective evaluation of the identified clusters has a major role until a desired or appropriate interpretation of the result is achieved. Also, as the size of Delphi data was rather small (*n* = 38) and the methodological approach more qualitative, the hierarchical cluster analysis was selected over *K*-means clustering. Varho and Tapio [34] suggest 3–7 alternative solutions as long as the dendrogram or hierarchical tree plotted by the used software allows this. The critical question is the number of clusters. According to Varho and Tapio [34] too, few clusters might result in a rather unimaginative outcome, and too many clusters might either make it difficult to discern actual differences between future states or be too many for decision-makers to grasp.

**Fig. 3:**
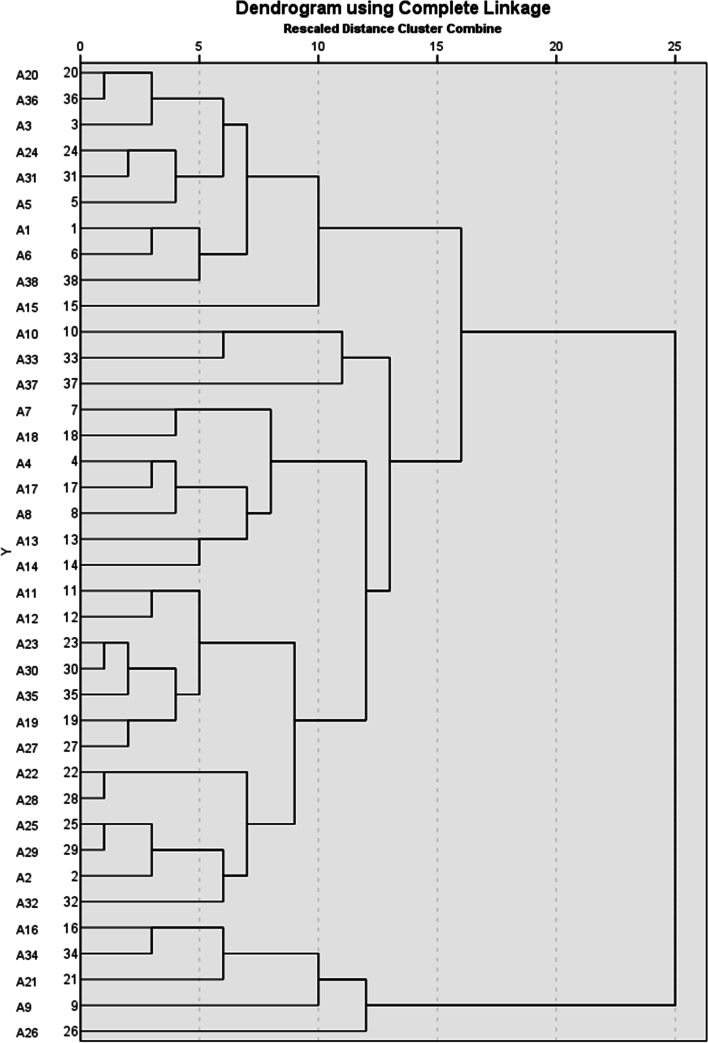
Dendrogram presenting the output from hierarchical clustering

After testing from a single solution to a range of three to eight solutions, it was noticed that taking the five-cluster solution or more would form one cluster with just one respondent. Therefore, we abandoned the solutions starting from five clusters and concentrated on the solutions of three and four clusters. Also, the four-cluster solution forms one cluster of three respondents on stage 12 (see Fig. 3 and Additional file 1: Appendix 1). We scrutinised this further, and when looking the differences in answers compared to cluster 2 from where cluster 4 gradually is composed, it can be seen that the main differences apply to statements 19 and 23. These statements pinpoint the importance of looking after domestic production, and therefore, it was considered that cluster 4 would not bring any interpretive value added to the analysis of the three-cluster solution (see Table 1 for statements 19 and 23). After these testing, the cluster run of three clusters was decided to form the basis for further analysis and interpretation of the resilience statements. The cluster centres were calculated as the arithmetic means of each variable within a cluster in addition to the values of standard deviation.

**Table 1:** Statements and three cluster centres related to determinants, drivers, and changes of a resilient food system

**Bold font = divergent view of the question** *Cursive font* = *parallel view of the question but includes some difference* Normal font = consensus view of the question	Cluster centre 1: **Lacking efficient and consensual global crisis preparedness (mean value [SD])a**	Cluster centre 2: **Looking after domestic food production (mean value [SD])a**	Cluster centre 3: **Trusting the current good resilience-building efforts (mean value [SD])a**
**Statement 1: Poor economic viability of primary production hinders the implementation of preparatory measures for resilience**	**0.9 [1.1]**	**1.0 [0.5]**	** − 0.6 [1.1]**
Statement 2: Poor economic viability of the food industry hinders the implementation of preparatory measures for resilience	** − **0.3 **[0.9]**	** − **0.2 **[0.8]**	** − **0.2 **[1.3]**
Statement 3: Poor economic viability of trade hinders the implementation of preparatory measures for resilience	** − **1.5 **[0.7]**	** − **1.1 **[0.9]**	** − **1 **[1.2]**
**Statement 4: The costs of promoting resilience are evenly distributed in the food system**	** − 1.4 [1.1]**	** − 0.4 [1.5]**	**1 [0.7]**
*Statement 5: Independence from imported inputs is currently sufficient from a resilience perspective*	** − ***1.7***[0.5]**	** − ***1.3***[0.8]**	** − ***0.4***[1.3]**
**Statement 6: Dependence on import of inputs does not jeopardise food security in crisis situations**	** − 1.7 [0.9]**	− **1.2 [0.7]**	**0 [1.4]**
Statement 7: Increasing the protein crop production area from the current level would improve resilience	1.5 **[0.5]**	1.5 **[0.5]**	1.4 **[0.5]**
**Statement 8: The production capacity for livestock production within agriculture can be increased on demand**	− **0.2 [0.8]**	**0.1 [1.0]**	**0.4 [0.9]**
Statement 9: Increasing domestic renewable energy (RE) production from current levels would improve resilience	1.7 **[0.5]**	1.5 **[0.9]**	1.2 **[0.4]**
Statement 10: Backup power systems should be increased in electricity production in different parts of the food system	1.0 **[0.9]**	1.3 **[0.6]**	1 **[1.0]**
*Statement 11: In the food system, dependence on foreign labour is currently too high*	*1.1***[1.0]**	*0.5***[0.8]**	*0.2***[1.1]**
**Statement 12: Employees’ mental well-being is currently adequate in terms of resilience**	− **1.4 [0.8]**	− **0.4 [0.8]**	**0.8 [0.4]**
*Statement 13: Increasing biodiversity from the current level would improve resilience*	*1.5***[0.7]**	*1.3***[0.6]**	*0.4***[0.9]**
**Statement 14: In the food system, the enterprise structure (variation in size, production branch, specialisation, and diversified) is sufficiently decentralised and diverse in terms of resilience**	− **1.3 [0.7]**	− **0.7 [0.8]**	**0.8 [0.8]**
*Statement 15: Crop selection and crop rotations should be diversified to promote primary production resilience*	*1.8***[0.6]**	*1.2***[0.7]**	*0.6***[0.9]**
**Statement 16: In the food system, system-level understanding and management are currently adequate, enabling a rapid response, decision-making, and process changes if necessary**	− **1.7 [0.5]**	− **0.6 [0.8]**	**0 [1.0]**
**Statement 17: In my view, current reserve stocks ensure the adequacy of inputs and products in crisis situations**	− **1.3[0.7]**	− **0.5 [1.1]**	**0.4 [1.3]**
**Statement 18: Awareness and control are currently level to prevent disturbances (e.g. animal diseases, pests)**	− **0.2 [1.0]**	**0.4 [0.9]**	**0.8 [1.1]**
**Statement 19: Cooperation between food system operators is currently sufficiently open and trust building in terms of resilience**	− **1.8 [0.4]**	**0.0 [1.1]**	**1.2 [0.4]**
**Statement 20: The food system actor network is currently sufficiently extensive and versatile in terms of resilience**	− **0.6 [1.2]**	**0.1 [0.8]**	**1.8 [0.4]**
**Statement 21: Society and food system actors are prepared with adequate backup systems for crisis situations to maintain food security**	− **1.4 [0.5]**	− **0.5 [0.9]**	**0.2 [1.3]**
*Statement 22: Sufficient attention is currently paid to the protection of information systems*	− *1.3***[0.5]**	− *0.7***[0.9]**	− *0.4***[1.3]**
Statement 23: Greater involvement of citizens in food communities would improve resilience	0.7 **[0.9]**	0.7 **[0.9]**	0.2 **[1.1]**
Mean value of all 23 statements	− 0.33 [0.76]	0.09 [0.85]	0.43 [0.97]

^a^Likert scale: − 2 completely disagree, + 2 completely agree (values between 0.5. 2 interpreted as agreeing, 0.4 and − 0.4 interpreted as not agreeing or disagreeing, and − 0.5 and − 2 interpreted as disagreeing)

In the final statistical runs that represent the cluster run of three clusters, we used the furthest neighbour algorithm with the Euclidean distance measure in the analysis. The furthest neighbour algorithm belongs to agglomerative hierarchical clustering methods [30]. It has a good performance in comparative studies on various algorithms [35]. The method uses Euclidean distance measure, and the number of clusters is not predetermined by the researchers. The algorithm starts by dealing with each case separately and clustering the two closest units of observation. Then each unit of observation (or made cluster) is compared to the furthest unit, or ‘furthest neighbour’, of each already made cluster (see also [34]). Distance to the furthest neighbour is minimised. In the end, all units and clusters merge in one cluster. The formed clusters are presented in Table 1, and the dendrogram of the final cluster run is presented in Fig. 3. In cluster 1, there was a total of 10 respondents,in cluster 2, a total of 23,and in cluster 3, five.

Based on the cluster analysis, three clusters were identified; the cluster centres and the values of standard deviations were calculated (see Table 1). The formed clusters weighted the resilience determinants differently. The clusters were named by their posture and frame of focus regarding food system resilience as follows: (1) lacking efficient and consensual global crisis preparedness, (2) looking after domestic food production, and (3) trusting the current good resilience-building efforts. The differences between the clusters are presented below.

Hierarchical cluster analysis groups all responses into a smaller number of different clusters, simplifying the variance within the data. The comparison between clusters’ mean values shows the overall differences in viewpoints between these three clusters. Comparing the overall Delphi data to three clusters’ mean values show that the cluster 1 has the lowest value, i.e. representing rather negative viewpoint on current resilience questions (the mean value of statements in cluster 1 is − 0.33). The cluster 2 mean value is more neutral (the mean value of all statements is 0.09). Cluster 3 separates itself clearly having a mean value of 0.43. Therefore, it stands out as more positive stance on resilience determinants, drivers, and changes. As the clusters are not similar in size, the differences in clusters focus more on interpreting intra-cluster variation than on cross-cluster variation. Cluster 3 is by far the largest in terms of internal standard deviation (the mean value of the SD in all answers is 0.97) but on the other hand contains smallest number of respondents (five). The mean of standard deviation in cluster 1 is lowest (0.77) and in cluster 2 0.85.

### Cluster 1: Lacking efficient and consensual global crisis preparedness

This cluster includes those expert respondents who are concerned about overall resilience within the food system. They consider system-level understanding and management in the food chain to be currently inadequate, and this does not enable rapid responses, decision-making, and process changes if required. They see that the threats to the global operating environment are extensive. Dependence on imports of inputs especially jeopardises food security in crisis situations.

They also consider that a lack of sufficient backup systems remains. Part of this is that employees’ well-being is currently inadequate in terms of resilience. Furthermore, current reserve stocks do not ensure the adequacy of inputs and products in crisis situations. Insufficient attention is paid to the protection of information systems. Overall, the enterprise structure (variation in size, production branch, specialisation, and diversified) is insufficiently decentralised and diverse. This cluster also considers that cooperation between food system operators is currently insufficiently open, and trust building and effort sharing in the food chain are unevenly distributed between the actors.

### Cluster 2: Looking after domestic food production

Cluster 2 can be described as a milder version of the former cluster. It includes those expert respondents who are reasonably satisfied with the current domestic system but are concerned about the profitability and independence of primary production and its development nationally. Indeed, the poor economic viability of primary production hinders the implementation of preparatory measures for resilience. This cluster also emphasises independence from imported inputs; it is currently insufficient from a resilience perspective, and dependence on imports jeopardises food security in crisis situations. This cluster also pinpoints the importance of independence in the energy system and considers that increasing domestic renewable energy production from current levels would improve resilience. Simultaneously, backup power systems should be increased in electricity production in different parts of the food system.

### Cluster 3: Trusting the current good efforts in resilience

This cluster is the most positive in the current situation. The expert respondents attached to this cluster rely on the current networks and cooperation and believe that the costs of promoting resilience are evenly distributed between the actors. They trust the current market and policy environment. They consider that the cooperation between food system operators is currently sufficiently open and trust building in terms of resilience. Furthermore, the food system actor network is sufficiently extensive and versatile in its resilience. Awareness and control are also adequate to prevent disturbances (e.g. animal diseases, pests).

In contrast with the other two clusters, this cluster considers that the poor economic viability of primary production does not hinder the implementation of preparatory measures for resilience. The enterprise structure (variation in size, production branch, specialisation, and diversified) is also sufficiently decentralised and diverse in terms of food system resilience. They also consider that employees’ mental well-being is currently adequate level in its resilience.

It should be noted that of the 23 statements, three were close to each other in the calculated values of these three cluster centres—namely, statements 7, 9, and 10. These can be considered consensus topics among the food system expert community, and they are the following: (1) increasing the protein crop production area from the current level would improve resilience, (2) increasing domestic renewable energy production from current levels would improve resilience, and (3) the backup power systems should be increased in electricity production in different parts of the food system.

### The expert panel background in clusters

**Fig. 4:**
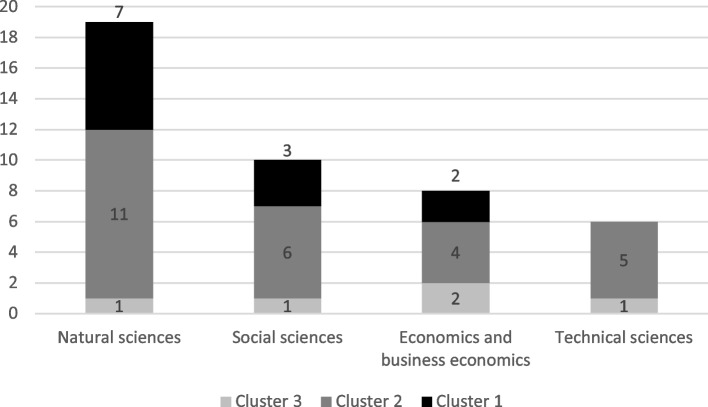
The educational background of the respondents (one respondent can have many educational backgrounds; *n* = 43; three of the educations not included in the figure, see Fig. 2)

**Fig. 5:**
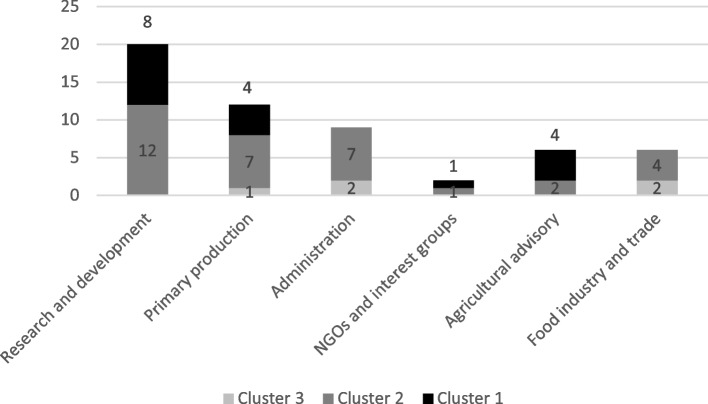
The actor and stakeholder status of respondents within the food system (one respondent can have many professional statuses; *n* = 55, one professional background not included in the figure, see Fig. 2)

Questions were also asked about the experts’ background questions, and they were utilised in analysing whether educational background or the respondent’s actor and stakeholder status within the food system explained the formed clusters (see Figs. 4 and 5). Educational background seems to deviate between the clusters quite evenly; only the technical sciences background is categorised in two of the formed clusters. It must be noted that the smallest number of respondents possessed a technical education—namely, six respondents. Cluster 3 included the most educational backgrounds from economics, business economics, and the technical sciences, whereas cluster 1 respondents came mainly from the natural and social sciences. Cluster 2 is more evenly distributed throughout all the educational backgrounds.

The actor and stakeholder background also deviated between clusters. Cluster 2 respondents were the most dispersed around the food system actors. In cluster 1, the respondents came from NGOs and interest groups and advisory, research, and primary production. Cluster 3 was formed by primary production, administration, and food industry and trade.

### The importance evaluation of the resilience of food system determinants

Following the rated questions, we also asked how important experts evaluated individual items from the resilience perspective. This was also asked in a Likert scale from one to five (not important at all—very important). Table 2 represents the five most important determinants attached to each of the clusters (if the mean value is the same, all the items are included).

**Table 2:** The five most important resilience items/questions/determinants (the bold text is used to highlight the item/issue/topic in question)

	Mean value^a^
**Cluster 1: Lacking efficient and consensual global crisis preparedness **(item/question/determinant)	
1. Increasing **domestic renewable energy (RE) production** from current levels would improve resilience	4.70
**2. Crop selection and crop rotations** should be diversified to promote primary production resilience	4.70
**3. Dependence on import of inputs** does not jeopardise food security in crisis situations	4.67
**4. The mental well-being of employees** is currently adequate in terms of resilience	4.67
**5. Poor economic viability of primary production** hinders the implementation of preparatory measures for resilience	4.60
6. Society and food system actors are prepared with adequate **backup systems for crisis situations** to maintain food security	4.60
**Cluster 2: Looking after the domestic food system** (item/question/determinant)	
**1. Poor economic viability of primary production** hinders the implementation of preparatory measures for resilience	4.48
**2. Independence from imported inputs** is currently sufficient from a resilience perspective	4.48
**3. Dependence on imports** of inputs does not jeopardise food security in crisis situations	4.43
**4. The costs of promoting resilience are evenly distributed** in the food system	4.39
**5. Increasing domestic renewable energy (RE) production** from current levels would improve resilience	4.35
6. **Cooperation between food system operators** is currently sufficiently open and trust building in terms of resilience	4.35
**Cluster 3: Trusting the current good efforts in resilience building** (item/question/determinant)	
**1. Cooperation between food system operators** is currently sufficiently open and trust building in terms of resilience	4.60
**2. The food system actor network is currently sufficiently extensive and versatile** in terms of resilience	4.60
3. Society and food system actors are prepared with adequate **backup systems** for crisis situations to maintain food security	4.60
**4. Dependence on imports of inputs** does not jeopardise food security in crisis situations	4.40
5. Sufficient attention is currently paid to the **protection of information systems**	4.40

^a^From one to five (not important at all—very important)

As Table 2 shows, cluster 1 considers the most important determinants for resilience as follows: (1) domestic RE production, (2) crop selection and crop rotations, (3) dependence on imports of inputs, (4) employees’ mental well-being, (5) the poor economic viability of primary production, and (5) (with the same value) backup systems for crisis situations. Cluster 2 highlights ‘the poor economic viability’ and ‘independence from imported inputs’ compared to cluster 1. ‘Domestic RE’ is also in the top 5 list but is lower than in cluster 1. Cluster 2 includes ‘the even cost distribution of promoting resilience in the food system’ and ‘cooperation between food system operators’ as different determinants.

Cluster 3 has ‘the cooperation between food system operators’ as the most important determinant, followed by ‘the extensive and versatile food system actor network’, which that is not in the top 5 list of the other clusters. ‘Protection of information systems’ is also a different determinant than in the former two clusters. ‘Dependence on imports’ can also be found in cluster 3, and the fifth determinant is ‘backup systems for crisis situations’.

When examining the Delphi expert panellists’ views of the overall importance (Table 3), the mean values in the top 5 resilience determinants are somewhat lower, and the list differs from clusters 1, 2, and 3. The dependence on the import of inputs can be found in each of the clusters, and it can be considered the main concern within the food system expert community. The poor economic viability and cooperation between food system operators can be found in two of the clusters, and domestic renewable energy (RE) production and evenly distributed costs of promoting resilience are found in one of the clusters.

**Table 3:** The five most important resilience items/questions/determinants in the whole Delphi panel (the bold text is used to highlight the item/issue/topic in question)

Resilience item/question/determinant	Mean value
**1. Dependence on import of inputs** does not jeopardise food security in crisis situations	4.49
**2. Poor economic viability of primary production** hinders the implementation of preparatory measures for resilience	4.39
**3. Cooperation between food system operators** is currently sufficiently open and trust building in terms of resilience	4.39
**4. Increasing domestic renewable energy (RE) production** from current levels would improve resilience	4.37
**5. The costs of promoting resilience are evenly distributed** in the food system	4.34

### Enablers and barriers from techno-economic, political-institutional, and socio-cognitive perspectives

During the Delphi survey, a respondent could argue with open-ended answers what kind of solutions, enablers, challenges, or barriers there were concerning their numerical evaluation. From these open-ended answers, Table 4 represents the results of these questions.

**Table 4:** Expert views of enablers and barriers for food system resilience. Results of open-ended questions

	**Techno-economic**	**Politico-institutional**	**Socio-cognitive**
Enablers	◾ Adjusting trade agreements ◾ Changes in taxation ◾ Changes in the farm subsidy system ◾ Increasing profitability of agricultural production ◾ Diversification of cropping ◾ Increased resources ◾ Domestic energy production ◾ Decentralised solutions	◾ Creation of clear criteria for resilience and monitoring systems to support them ◾ Supporting policy measures ◾ Systematic framework for resilience (system thinking) ◾ Political will ◾ Enhanced communication and swift information flows between different sectors	◾ Establishing targets ◾ Changes in attitudes ◾ Sense of fairness ◾ Collaboration and trust between actors in the food system ◾ Increased awareness ◾ Functional examples
Barriers	◾ Means to increase resilience are costly, but do not transfer into added income ◾ Using mainly demand-based measures ◾ Poor profitability ◾ Current subsidy system does not encourage increased resilience ◾ Disconnections between production and its inputs ◾ Unreasonable trust in market forces ◾ Lack of economic drivers and interests ◾ Small volume of the domestic markets	◾ Lack of a bigger picture ◾ Fragmentation of the components regarding resilience ◾ Lack of scientific knowledge ◾ Stiffness and inertia at the political level ◾ Current political climate and lack of will ◾ Trends in the globalised food system counteract increasing resilience at the national level ◾ Overall system gives wrong signals for increasing resilience ◾ Corruption	◾ Sense of unfairness ◾ Lack of understanding ◾ Poor level of organisation within the food system ◾ Reluctance and conflicts of interest ◾ Established attitudes and practices ◾ Lack of motivation

The open answers (Table 4) provided an insight into various system aspects the respondents deemed important. First, the techno-economic orientation of the current food system in Finland was found incompatible with reinforcing the system’s resilience. The poor profitability of agricultural production, stemming from the sluggish subsidy system, currently failed to provide reasonable incentives for added resilience. Second, it was argued that the globalisation of the food system created fragmentation, which hindered the ability to systematically assess all the relevant interactions and domains required to build system resilience. In some cases, it was argued that the global trends even counteracted the recognised means to build a more resilient system at the national level. Third, it was found that the political framework emphasised market-oriented and demand-centric means in food policy, which was manifested as the poor understanding and subsequent coordination of the resilience theme. It was argued that a lack of systematic resilience thinking, combined with the political system’s stiffness and inertia, gave mixed signals for the food system actors, yielding sluggish progress at best. Finally, actors within the food system could be reluctant and unmotivated to engage in actions to enhance resilience. It was argued that this stemmed from a lack of understanding, conflicting interests, established attitudes, or a sense of unfairness regarding development in the food system dynamic.

To tackle these issues, the respondents proposed a more systematic framework for food system resilience. A common framework would highlight the variety of actors and policy dimensions involved when reinforcing food system resilience, creating a more cohesive set of means to address it. Additionally, clear targets and criteria for resilience should be created for food system actors and policymakers alike to track progress in the matter. Better communication and collaboration between actors in the food system could generate added trust between the actors and a broader understanding of food system resilience. In turn, this could reduce reluctance to act and create a motivation to participate in building a more resilient food system from the bottom up. Tangible functional examples would create willingness and lower the threshold for participation. At the political level, additional subsidies and other policy means were found important drivers to accelerate the development of a more resilient food system. The profitability of agricultural production should especially be emphasised, and costs related to increasing resilience compensated either through producer prices or by taxation. Domestic decentralised solutions in both agricultural production and energy production were found to support resilience in Finnish conditions.

## Discussion

In this paper, we have analysed the key determinants of a resilient food system using an expert method. The study therefore forms a practice-oriented view of the food system actors’ views and argumentation in making food systems more resilient to confront changes. An analysis of the expert process result showed the different weights of the key priorities for the development of a resilient food system. The three experts’ opinion polls formed by cluster analysis pinpointed the following: (1) the lack of efficient and consensual global crisis preparedness; (2) concerns of the domestic food system, especially the poor profitability and its effect on resilience building; and (3) a more optimistic view and trust in current resilience-building efforts, especially pinpointing the importance of tight cooperation between food system actors.

According to the results, the weakest point in the food system is primary production and its poor profitability in the eyes of resilience building. The current structural development of primary production, resulting in a concentration of production sectors in different areas of Finland, accelerated by the subsidy system, fails to provide reasonable incentives for resilience development (e.g. nutrient cycling). It was argued that a lack of systematic resilience thinking, combined with the political system’s stiffness and inertia, gave mixed signals for food system actors, yielding at best sluggish progress in building resilience. A tangible example is the structural development of farms so far. The development in the last 25 years has meant that there has been little variation in size, production branch, specialisation, and diversification on farms than was the situation, e.g. in 1995, when Finland joined the EU [5]. It can be also noted that the key reasoning behind the development was the economies of scale, and there has yet to be much discussion of whether the Finnish farm structure is sufficiently decentralised and diverse in terms of resilience.

Clear targets and criteria for resilience were considered important, and these should be created for food system actors and policymakers alike to focus on the right things and track progress in the matter. Improving communication and collaboration between actors in the food system could generate added trust between them and a broader and systemic understanding of food system resilience. There was a strong consensus among the food system expert community on the three main areas of resilience building. First, increasing the protein crop production area from the current level would improve resilience. Second, increasing domestic renewable energy production from current levels would also improve resilience. Third, the backup power systems should be increased in electricity production in different parts of the food system.

The three clusters formed reflect the differences in weighting the resilience factors rather than being completely disconnected and exclusive from each other. They therefore differ in their framing of food system resilience and in the trends and forces that contribute to a more resilient system. Cluster 1 appears to strongly emphasise the structure of Finnish food production as part of the global food system and calls for strong public policy to steer resilience development. It approaches the food system from a structural perspective and seems more cautious about the structural developments in the system compared to other clusters. This cluster also seems the most concerned about the actors within the food system, calling for attention to the poor developments in actors’ mental well-being, cooperation between actors, and the equal distribution of the burden between food system actors. Cluster 2 appears more concerned about the functionality of the domestic food system and especially the continuity and independence of domestic food supply. This cluster especially focuses on the poor economic conditions in primary production and advocates solutions targeted mostly at improving the operating conditions and self-sufficiency of inputs in production to enhance the overall food system’s resilience. Finally, cluster 3 seems to emphasise the role of undisturbed market forces and a clear continuous political framework as the optimal way to ensure resilience in the food system. In this view, resilience could be promoted through market selection, in which competitive and capable enterprises would recognise the importance of resilience in their operations and organically seek to reduce the impact of potential disturbances through planning and increased cooperation with other food system actors. The answers in this cluster portray the current state of food system resilience significantly more positively and seem to accept creative destruction and market orientation in the food system more willingly than the other clusters.

To build society’s resilience and adaptive capacity for change, anticipation of future challenges can provide a common insight, both in confronting short-term distortions and building long-term adaptation strategies. This can be improved by defining and sharpening the foci of current food system foresight. The public discussion of the COVID-19 pandemic may lead to a sudden food crisis if the pandemic is prolonged [3]. A food crisis can fundamentally affect basic social security and create chaos, even in peaceful societies. National food system foresight should therefore be targeted to assist societal decision-making in sustaining and improving resilience and adaptive capacity in food systems.

One possibility from the resilience perspective is to generate an indicator setup for the use of follow-up, anticipation, and evaluation. As resilience calls for a holistic framework to identify the main vulnerabilities and determinants, i.e. weak and strong points within the food system, a well-balanced indicator setup could be a practical tool to monitor and anticipate the big picture development. Engle et al. [36] propose a framework for developing indicators that analysts might select as useful for guiding future development and adaptation decisions. Cordoba et al. [37] emphasise the importance of including and considering the biophysical variables, management practices, structure, and agency of agriculture through a participatory approach. The capacity of agency received a greater weight, especially in cluster 3, in our overall evaluation of resilience determinants. A good start to building a systematic monitoring setup can be an expert process in which the food system expert community defines the key monitoring needs and indicators. Indicators should capture perspectives of both resistance and recovery at multiple spatial and temporal scales [38]. Our study has shown that especially the monitoring of profitability, domestic renewable energy production, crop diversity, including both crop selection and the level of crop rotations in production, and dependence on the import of inputs were considered very important by the food system experts. Indicators for monitoring employees’ mental well-being within the food system, the evaluation of the current and future needs of backup systems, the level of cooperation and trust between food system operators, the extensiveness and versatility of the food system actor network, and the level of the protection of information systems should also be found.

For some time now, especially with the COVID-19 pandemic, system resilience has received more attention. Engle et al. [36] define resilience as the potential to absorb and cope with the impacts of short-term climate shocks and extremes and to learn, reorganise, and redevelop, preferably to an improved state, in the longer term. Strategies for building resilience combine preparedness for an immediate response to extreme events with long-term sustainable development objectives that increase the socioeconomic and environmental capacity to function in new climate conditions. More sustainable production systems are therefore needed to improve agricultural productivity while protecting natural ecosystems and safeguarding their important functions. This issue has been highlighted in several policy documents and initiatives such as the UN Sustainable Development Goals [39], the European Green Deal [40], the Farm to Fork Strategy [41], the EU Biodiversity Strategy [42], the CAP [43], and the National Food Strategy in Finland [44]. These new visions and strategies are sufficiently bold to embrace the transitions in the food system, but they are somewhat lacking in a resilience perspective. Sustainability, environmental concerns—especially water protection—and biodiversity loss in current production systems have received most attention. However, if the visions and strategies for developing resilience are not well grounded in the present, they fail to take necessary actions into account. Moreover, the costs of developing resilience should be acknowledged, based on the shared understanding of the food system dynamics and the development needs. To achieve a more resilient food system, we need a thorough knowledge of the determinants of resilience, as well as of the environmental, economic, and sociocultural impacts of various actions. Finally, we need to engage with actors at different levels of society to accomplish the required transition.

## Conclusions and policy implications

In this study, we analysed through the Delphi method three key questions about food system resilience. Firstly, we analysed what are the key priorities for the development of a resilient food system. According to the Delphi study results, the weakest point from the food system resilience perspective is primary production and its poor profitability. The structural development of farms has been rapid in the last 25 years, and the aim has been to invest in growth and specialisation among farms. At the same time, a regional concentration of agricultural production has occurred. To maintain and develop resilience, agricultural and other policies that guide food production should consider and evaluate the vulnerabilities of such a rapid structural development. The Delphi panel agreed on three key issues: resilience can be developed by increasing the protein crop production in agriculture, by increasing domestic renewable energy production, and by increasing the backup power systems in electricity production in different parts of the food system.

Secondly, we also analysed how do key priorities and weights vary among the food system expert community. According to the results, there are various means to sustain and increase resilience in which the expert community weights are more diverse, e.g. the dependence on import of inputs and increasing domestic renewable energy production. The self-sufficiency and security of supply of agricultural production should therefore play a strong role in agricultural policy measures during the 2020s. The efficient implementation of the EU’s Green Deal [40] and the Farm to Fork Strategy [41] can support this green transition, especially in renewing the energy system in agriculture.

Thirdly, we analysed what are the enablers promoting and barriers hampering the resilience of the Finnish food system. The results indicate that collaboration between food system actors increases trust and the systemic understanding of food system development. Based on the sudden shocks and disruptions, clear targets and criteria for food system resilience should be created, and resilience should be included in the strategies that guide food system development throughout the different parts of the food chain. As a main barrier, the experts considered the lack of systematic resilience thinking, combined with the political system’s stiffness and inertia as the main barrier can give mixed signals for the food system actors, yielding sluggish progress at best. The public authorities and key food system operators should continue their tight collaboration, especially in the current organisation for the security of supply. Through this, trust in and systemic understanding of food system development will increase.

## Supplementary Information

**Additional file 1:** Proximity matrix.

## Data Availability

The Delphi process data was gathered by the researchers in the Natural Resource Institute Luke (organised by Professor Pasi Rikkonen) and stored in the databases of the Luke under a password (including backup data in the organisation server). Luke was responsible for taking care of the registry containing identifying information of the participants and the Privacy Notice of Processing Personal Data. The datasets generated during the Delphi process are available from the corresponding author when the project is over on reasonable request. Data will be anonymised.
