# Supplementary figures and images for: The determinants of a resilient food system for Finland in the 2020s—three opinion polls for improvements based on a Delphi study among food system experts
Source: Eur J Futures Res. 2023 Mar 13;11(1):2. doi: 10.1186/s40309-023-00215-z (PMC10008712; doi:10.1186/s40309-023-00215-z)

Appendix 1:


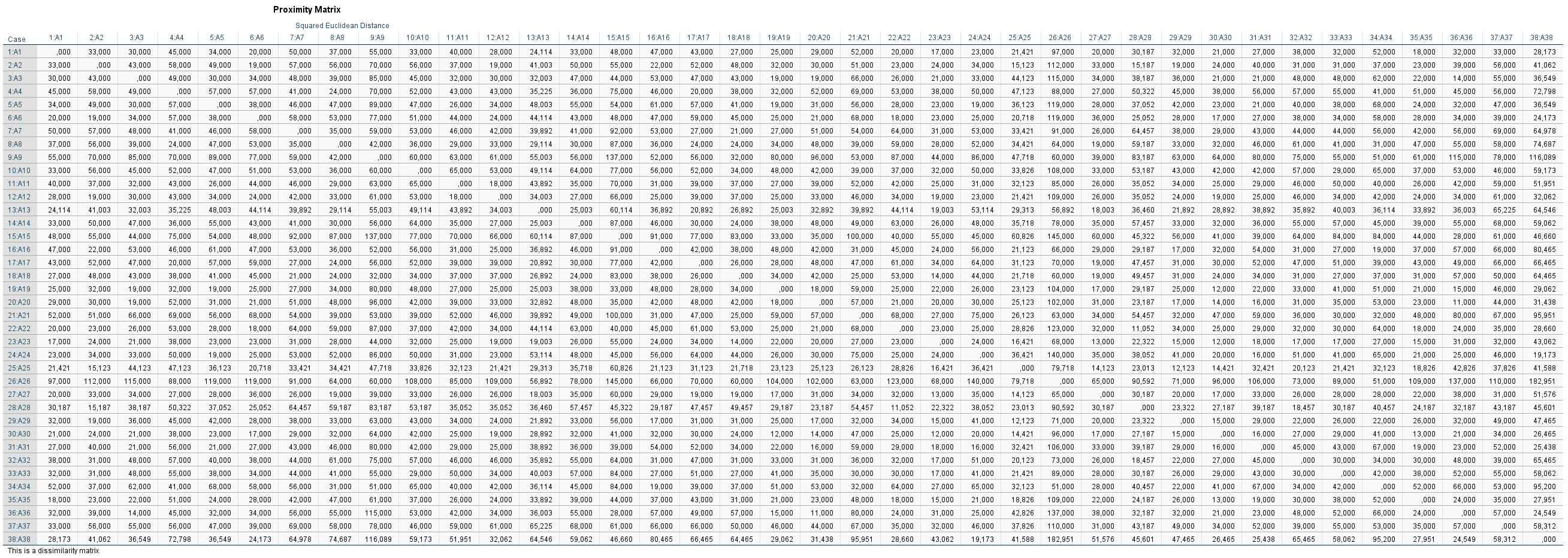

Supplement: Supplementary file 1 — Additional file 1: Proximity matrix. [file 40309_2023_215_MOESM1_ESM.docx]
